# Correlation Between Immune-Related Genes and Tumor-Infiltrating Immune Cells With the Efficacy of Neoadjuvant Chemotherapy for Breast Cancer

**DOI:** 10.3389/fgene.2022.905617

**Published:** 2022-06-08

**Authors:** Yan Zhou, Qi Tian, Huan Gao, Lizhe Zhu, Jiao Yang, Juan Zhang, Jin Yang

**Affiliations:** ^1^ Department of Medical Oncology, The First Affiliated Hospital of Xi’an Jiaotong University, Xi’an, China; ^2^ Department of Breast Surgery, The First Affiliated Hospital of Xi’an Jiaotong University, Xi’an, China

**Keywords:** tumor immunology, immunogenetics, tumor immune microenvironment, biomarkers of breast cancer, neoadjuvant chemotherapy

## Abstract

**Background:** In the absence of targeted therapy or clear clinically relevant biomarkers, neoadjuvant chemotherapy (NAC) is still the standard neoadjuvant systemic therapy for breast cancer. Among the many biomarkers predicting the efficacy of NAC, immune-related biomarkers, such as immune-related genes and tumor-infiltrating lymphocytes (TILs), play a key role.

**Methods:** We analyzed gene expression from several datasets in the Gene Expression Omnibus (GEO) database and evaluated the relative proportion of immune cells using the CIBERSORT method. In addition, mIHC/IF detection was performed on clinical surgical specimens of triple-negative breast cancer patients after NAC.

**Results:** We obtained seven immune-related genes, namely, *CXCL1, CXCL9*, *CXCL10*, *CXCL11*, *IDO1*, *IFNG*, and *ORM1* with higher expression in the pathological complete response (pCR) group than in the non-pCR group. In the pCR group, the levels of M1 and γδT macrophages were higher, while those of the M2 macrophages and mast cells were lower. After NAC, the proportions of M1, γδT cells, and resting CD4 memory T cells were increased, while the proportions of natural killer cells and dendritic cells were decreased with downregulated immune-related genes. The results of mIHC/IF detection and the prognostic information of corresponding clinical surgical specimens showed the correlation of proportions of natural killer cells, CD8-positive T cells, and macrophages with different disease-free survival outcomes.

**Conclusion:** The immune-related genes and immune cells of different subtypes in the tumor microenvironment are correlated with the response to NAC in breast cancer, and the interaction between TILs and NAC highlights the significance of combining NAC with immunotherapy to achieve better clinical benefits.

## Introduction

Neoadjuvant systemic therapy (NST) has become one of the major preoperative treatments for all subtypes of breast cancer (BC) over the past decade (Steenbruggen et al., 2017). Although chemotherapy in combination with other treatments is currently the preferred approach in NST, neoadjuvant chemotherapy (NAC) remains the standard NST in the absence of targeted therapy or clear clinically relevant biomarkers (Lebert et al., 2018). The therapeutic effect of NST in early BC can be reflected by some indicators, such as pathological response rate and disease-free survival (DFS) (Kong et al., 2011; Vaidya et al., 2018).

No specific methods have been established to predict the treatment response of patients receiving NAC; therefore, many predictors have been proposed to predict the prognosis of BC patients and select appropriate treatment strategies (Xu et al., 2020). In addition, multi-omics analysis has become a burgeoning approach to identify solid tumors for different molecular characteristics and clinical outcomes in recent years (Su et al., 2020). Among many predictors of whether patients achieve pathological complete response (pCR) and DFS benefit, tumor-infiltrating lymphocytes (TILs) and immune-related genes play a key role (Chica-Parrado et al., 2020), which is not only related to the crucial role of the tumor immune microenvironment (TIME) in the antitumor process but also related to the modulatory effect of chemotherapy on the TIME in NAC. The role of the TIME in the antitumor process can be determined by the correlation between the TIME and NAC response, whereas the modulation effect of chemotherapy on the TIME can be interpreted from the changes in the TIME before and after NAC.

Several researchers have made an effort to predict the NAC response by immune-related genes and obtained a prediction model with several genes as the core (Pérez-Pena et al., 2019). In addition, the changes in immune-related genes before and after NAC have also been summarized (Di Cosimo et al., 2019), but there is still no acknowledged NAC response–related immune gene set in BC. In terms of the TIME, TILs have been widely recognized as a crucial biomarker for predicting the efficacy of NAC in BC for a long time, which has been highlighted by a few pooled analyses, especially in triple-negative breast cancer (TNBC) (Loi et al., 2019; Park et al., 2019). In addition, changes in TILs before and after NAC are related to the therapeutic effects and relatively long-term prognosis in addition to pCR rate (such as DFS) of BC patients (Hamy et al., 2019; Herrero-Vicent et al., 2019). The relationships of various immune cell subtypes in TILs with the prognosis of NAC and its changes are serious and controversial topics of research (Kim et al., 2020).

In the present study, several datasets were used to analyze the role of immune-related genes and TILs in the therapeutic effect after NAC and their changes before and after NAC, which will identify several immune-related genes and corresponding immune cells related to the NAC response. In addition, we also used the clinical surgical specimens of BC patients after NAC to obtain the differences in the proportion of several types of immune cells to verify the correlation between different subtypes of TILs and prognosis.

## Materials and Methods

### Gene Expression and Clinical Data

High-throughput sequencing gene expression data (Illumina HiSeq 2500 format) from the GSE123845 dataset and microarray gene expression data (Affymetrix U133 Plus 2.0 gene expression array format) from four datasets (GSE32646, GSE31519, GSE32072, and GSE18728) were obtained from the National Centre for Biotechnology Information (NCBI) in the Gene Expression Omnibus (GEO) database. The GSE123845, GSE32646, and GSE31519 datasets were used to investigate the relationship between immune gene expression and response in patients who received NAC, while the GSE32072 and GSE18728 datasets were used to compare the changes in immune genes and immune infiltration before and after NAC. Detailed information about the datasets is available in Supplementary Table S1. Gene expression levels (mRNA expression z score from RNA-sequence) of 1097 BC patients from The Cancer Genome Atlas (TCGA) genomic cancer data were used to explore the relationship of seven differentially expressed genes (DEGs) with immune cell fractions.

Formalin-fixed paraffin-embedded (FFPE) tissue sections (4 µm thick) from six nonmetastatic TNBC patients admitted to the Department of Medical Oncology, The First Affiliated Hospital of Xi’an Jiaotong University during 2016–2020 were utilized in this study to verify the correlation between the immune cell proportion in TILs and the prognosis of TNBC patients who received NAC. DFS was defined as the time from surgery to the occurrence of the first metastasis, and the detailed clinical information, grouping, and treatment of patients are described in Table1.

**TABLE 1:** Detailed clinical information of six patients.

Sample No.	Age	Histological	Ki-67 (%)	T stage	N stage	NAC regimen	DFS (month)	Metastatic sites
1	35	Right breast nonspecific type of invasive cancer grade III flap intravascular cancer thromboembolus (MP2)	40	T2	N3	TEC∗2-NP∗2	5	Brain
2	46	Left breast invasive carcinoma (MP3)	40	T4d	N3	TAC∗3	5	Chest wall
3	60	Right breast nonspecific invasive ductal carcinoma (MP2)	80	T4a	N2	TE∗2—NP∗5	7	Liver
4	44	Right breast nonspecific invasive ductal carcinoma grade III(MP2)	80	T2	N1	TE∗6	23	Liver
5	38	Left breast nonspecific type of invasive cancer grade III	90	T3	Nx	TEC∗3	13	Lung
6	52	Right breast nonspecific type of invasive cancer grade II(MP2)	50	T2	N1	EC-T∗8	14	Multiple metastases (lymph nodes, two lungs, and bone)

TEC, docetaxel, epirubici, and cyclophosphamide; NP, vinorelbine and cisplatin/carboplatin; TAC, docetaxel, doxorubicin, and cyclophosphamide; TE, docetaxel and epirubici; EC-T, epirubici/cyclophosphamide followed by paclitaxel.

### Identification of DEGs, Gene Ontology , and Kyoto Encyclopedia of Genes and Genomes Pathway Enrichment Analysis of DEGs

DEGs were extracted and analyzed using the Limma, Impute, and EdgeR packages in R (Ritchie et al., 2015; McDermaid et al., 2019). DEGs were defined as *p* < 0.05 and log of fold change (logFC) > 1 or logFC ≤ −1. In the GSE123845 and GSE32646 datasets, the pCR groups were set as the control groups, and the non-pCR groups were set as the experimental groups. In the GSE32072 and GSE18728 datasets, the pre-NAC groups were set as the control groups, and the post-NAC groups were set as the experimental groups. Gene Ontology (GO) analysis refers to the high-throughput annotation of biological functions (BP), cellular components (CC), and molecular function (MF) of all genes in the genome by using bioinformatics methods and tools. The Kyoto Encyclopedia of Genes and Genomes (KEGG) (https://www.kegg.jp/) (Kanehisa et al., 2017) is a database that provides gene and genome functional significance at the molecular and pathway levels. The DOSE, ClusterProfiler, Org.Hs.eg.db, and Enrichplot packages in R (Yu et al., 2012; Yu et al., 2015; Pu et al., 2021) were used for the GO and KEGG pathway enrichment analyses of DEGs.

### Gene Set Enrichment Analyses

GSEA was performed to compare the gene expression among hallmark gene sets of pCR and non-pCR patients using GSEA version 4.1.0 provided by the Broad Institute (http://software.broadinstitute.org/gsea/index.jsp) (Subramanian et al., 2005). KEGG pathway enrichment analysis was performed using gene sets from the Broad Institute (http://ftp.broadinstitute.org://pub/gsea/gene_sets/c2.cp.kegg.v6.2.symbols.gmt). In GSEA, the significance of each pathway was classified by a threshold of false discovery rate (FDR) q-value <0.05.

### Protein–Protein Interaction Network Construction and Network Core Genes Extraction

To explore interactions, including gene neighborhood, fusion, and co-occurrence of DEGs, Search Tool for the Retrieval of Interacting Genes/Proteins (STRING) version 11.0 (Szklarczyk et al., 2019) was used by inputting the gene names of the DEGs and exporting the results with a minimum required interaction score of 0.7 as the PPI network. Genes with a number of adjacent nodes ≥7 were identified as network core genes.

### CIBERSORT Deconvolution Algorithm

The CIBERSORT deconvolution algorithm was used to estimate the fraction of 22 immune cell types in each tumor tissue to evaluate intratumor immune cell composition (Newman et al., 2015). These 22 cell fractions were calculated *via* the online calculator (https://cibersort.stanford.edu/) as previously reported (Takeshita et al., 2019). The fraction of 22 immune cell types estimated from each sample was filtered by *p* value < 0.05 to obtain more accurate prediction results.

### Survival Analysis

For plotting Kaplan–Meier (K-M) curves of GSE31519 grouping samples based on the proportion of different immune cells, GraphPad Prism 8.0.0 for Windows (GraphPad Software Inc., San Diego, California, USA) was used, and the log-rank test was used to assess the significance of event-free survival (EFS) differences.

### Multiplex Immunohistochemistry/Immunofluorescence Protocol and the Preparation of Fractions of Different Immune Cells and Tumor Cells

To identify tumor cells and different subsets of immune cells in the TIME, immunofluorescence staining was performed using the Pano 7-Plex IHC kit (Cat. No. 0004100100; Panovue, Beijing, China), according to previously published methods (Taube et al., 2020; Yeong et al., 2020). In brief, slides were incubated with different primary antibodies (CD56, CD8, CD68, HLA-DR, and PanCK) followed by incubation with horseradish peroxidase–conjugated secondary antibodies and tyramine signal amplification. The slides were treated with microwave heat treatment after each TSA (PerkinElmer, Waltham, Massachusetts, US) operation. After all human antigens were labeled, the nuclei were stained with 4′-6′-diamidino-2-phenylindole (DAPI, Sigma–Aldrich). To obtain a multispectral image, the colored slides were scanned using a Mantra system (PerkinElmer, Waltham, Massachusetts, US), which captured fluorescence spectra at 20-nm wavelength intervals from 420 to 720 nm at the same exposure time and combined them into a single stack image.

The autofluorescence spectra of tissues and each luciferin were extracted from unstained and single-stained section images. The extracted images were further used to establish the spectral library required for multispectral decomposition through inForm image analysis software (PerkinElmer, US). Using this spectral library, we obtained reconstructed images without self-fluorescence. The fluorescence score (Hscore value) of each antigen stain and the fractions of different immune cells and tumor cells in the TIME were also obtained using the software.

### Statistical Analysis

The statistical analyses of the data from the various datasets were performed using R software (http:///www.r-project.org/) and Bioconductor (http://bioconductor.org/) (Sepulveda, 2020). For differential gene analysis between groups, the Wilcoxon test was conducted using R software. To explore the association of 21 immune cell subtypes between the control groups and experimental groups, the Wilcoxon test was used using R software. A heatmap was produced with the pheatmap package in R (Pu et al., 2021). The nonparametric independent sample t-test was used to examine the difference in the fraction of cells between the long-DFS and short-DFS groups. All *p* values were bilateral, and a *p* value of <0.05 was considered statistically significant.

## Results

### BC Patient Response to NAC Is Related to Immune-Related Genes

**FIGURE 1:**
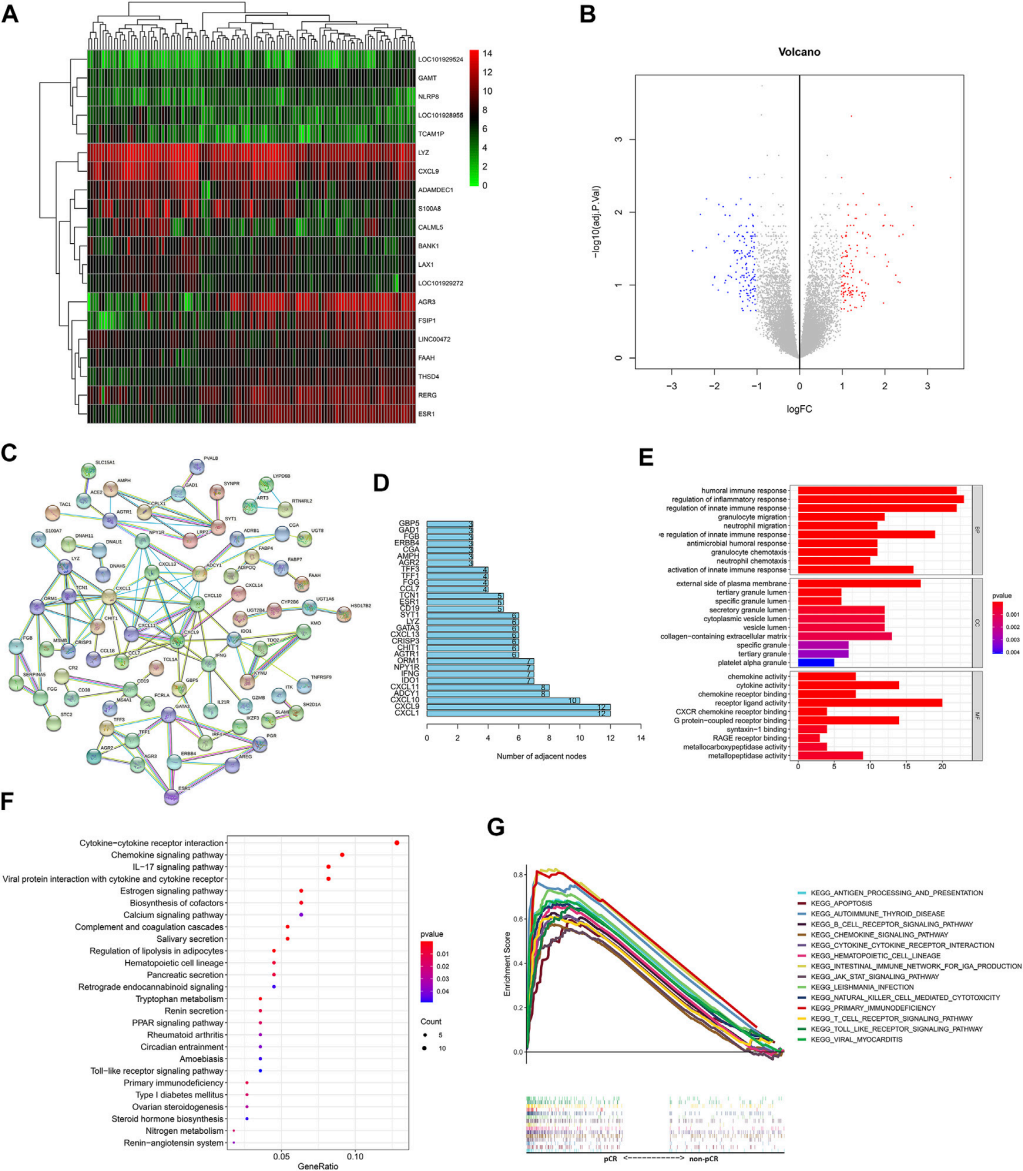
Response of BC patients to NAC is related to immune-related genes and pathways. **(A)** Genetic clustering analysis of the heatmap showed DEGs (*p* value < 0.05, logFC >1 or logFC < −1) with the fold change TOP20 from dataset GSE32646. The redder points in the heatmap indicate the higher expression of the gene in the corresponding sample, while the green points indicate the lower expression. **(B)** Volcanic diagram of gene expression from the dataset GSE32646. All genes are in the diagram, the red dots represent logFC >1 and *p* value < 0.05 DEGs; the blue dots represent logFC < −1 and *p* value < 0.05 DEGs; the rest were gray points with no statistical significance. **(C)** PPI results obtained from DEGs of dataset GSE32646. Each node represents a gene and the line between the circle nodes represents the interaction between the two proteins. **(D)** Number of adjacent nodes for each gene is shown in a bar chart and genes with number of adjacent nodes ≥7 were identified as network core genes. **(E)** DEGs from dataset GSE32646 are enriched in the GO containing BP, CC, and MF. **(F)** KEGG pathways of DEGs from the dataset GSE32646. One bubble represents a KEGG term, the size of the bubble represents the number of genes in the enriched signaling pathway, and the color represents significance. **(G)** Visualization results of the KEGG pathways with NES value TOP15 obtained from the gene expression data in the dataset GSE123845.

In total, 27 pCR samples and 88 non-pCR samples from the GSE32646 dataset were used for differential gene expression analysis, which identified 302 DEGs (filtering with *p* = 0.05 and logFC = 1; Supplementary Table S2). Genetic clustering analysis of the heatmap shows the top 20 genes according to the absolute value of the fold change (Figure 1A), and a volcano plot of the DEGs is shown in Figure 1B. The PPI results obtained from the abovementioned DEGs are shown in Figure 1C, which had a high confidence result with 0.7 as the minimum required interaction score and the removal of the independent gene. Using the number of adjacent nodes as criteria, we obtained the network core genes, including *CXCL1*, *CXCL9*, *CXCL10*, *ADCY1*, *CXCL11*, *IDO1*, *IFNG*, *NPY1R*, and *ORM1* (Figure 1D), and the immune-related genes included several chemokine CXC subfamily molecules, namely, *IDO1*, *IFNG*, and *ORM1*.

In addition, 1097 BC samples from the TCGA database were divided into two groups with high and low expression based on the immune-related DEGs extracted from the GSE32646 dataset. The differential analyses of the 22 immune cell fractions are shown in Supplementary Figures S1A–E. The differences in the expression levels of *CXCL1*, *CXCL9*, *IDO1*, and *IFNG* between the high- and low-expression groups showed that M1 macrophages, CD8 T cells, and resting CD4 memory T cells accounted for a high proportion in the high-expression group and that M2 macrophages and resting mast cells accounted for a high proportion in the low-expression group. The trend of differences for *ORM1* between groups was the same as that for the abovementioned DEGs, but the differences were not statistically significant.

### BC Patient Response to NAC Is Related to Immune-Related Pathways

GO enrichment analysis of the GSE32464 dataset showed that the BPs of the DEGs were mainly enriched in the migration and chemotaxis of immune cells and immune response and the MF of the DEGs was enriched in the regulation of the activity of chemokines and their receptors (Figure 1E). In addition, KEGG pathway analysis indicated that the DEGs were enriched in cytokine–cytokine receptor interactions, chemokine signaling pathways, and IL-17 signaling pathways (Figure 1F).

In addition, the gene expression data in the GSE123845 dataset were divided into the pCR group and non-pCR group, which were used to conduct GSEA to obtain the KEGG pathways with normalized enrichment score (NES), and the top 15 pathways are shown in Figure 1G. The signaling pathways were enriched in immune-related pathways, including the chemokine signaling pathway, apoptosis-related pathways, hematopoietic cell lineage–related pathways, and pathogen infection–related pathways.

### Differences Between Immune Cell Fractions of the Tumor Microenvironment in BC Patients Who Received NAC With Different Therapeutic Effects

**FIGURE 2:**
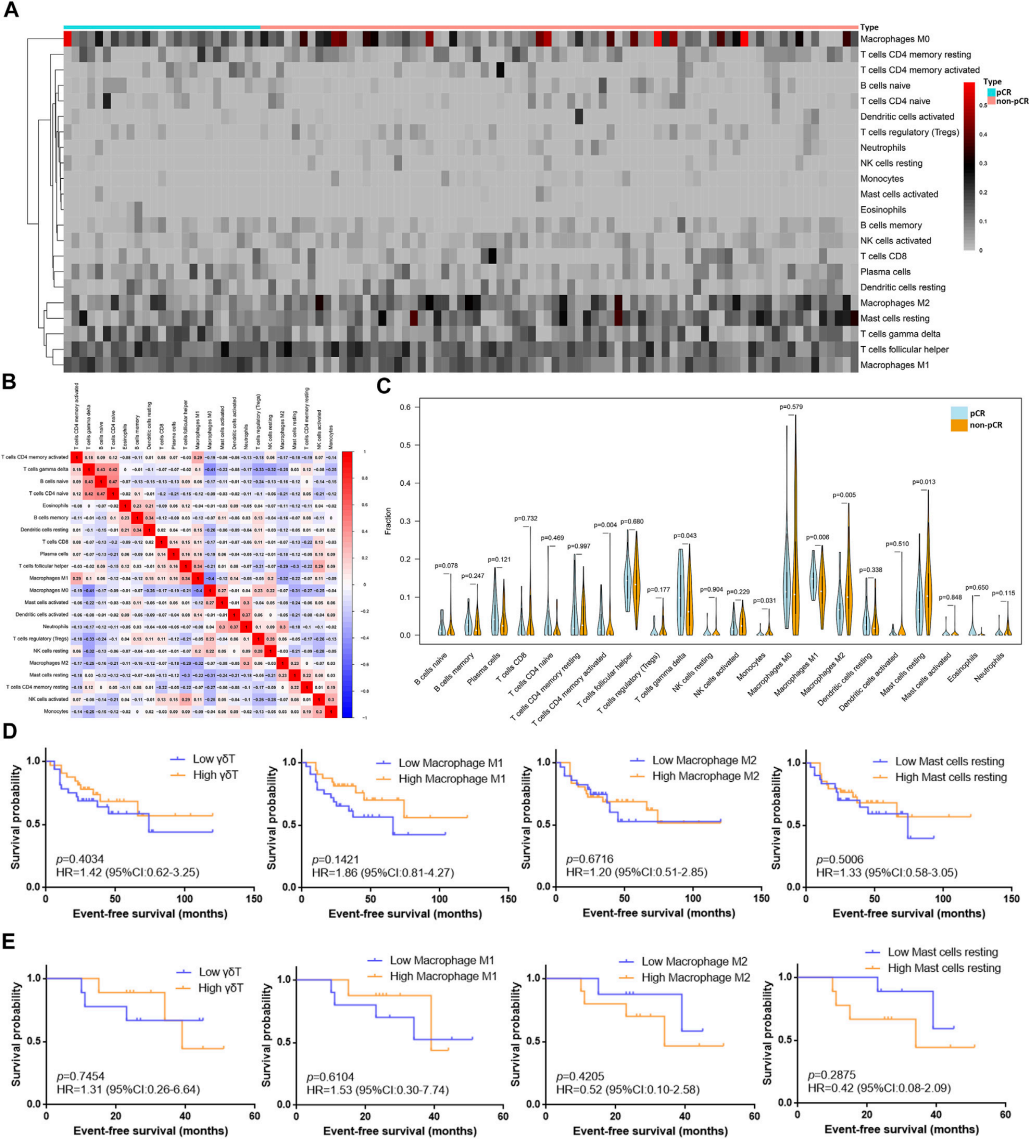
Differences between immune cell fractions of the TIME in BC patients with different NAC responses. **(A)** Heatmap of the fraction of 22 immune cell types for comparison between pCR samples and non-pCR samples from dataset GSE32646. The redder points in the heatmap indicate that the fraction of immune cells in the corresponding sample was higher, while the gray points indicate lower fraction. **(B)** Correlation of 22 immune cells in the TIME from dataset GSE32646: Red represents positive correlation, blue represents negative correlation, and the depth of color indicates the strength of correlation. **(C)** Differential analysis of 22 immune cell fractions in pCR and non-pCR samples from dataset GSE32646. Blue represents pCR samples and orange represents non-pCR samples, and *p* value represents the statistical significance of the differential analysis between the two groups for each immune cell. **(D)** Survival analysis of different grouping by immune cells subtypes in 63 TNBC samples from dataset GSE31519. **(E)** Survival analysis of different grouping by immune cells subtypes in 18 TNBC samples received NAC from dataset GSE31519.

The 22 immune cell type fractions obtained from the gene expression data of the GSE32646 dataset using the deconvolution algorithm are shown by a heatmap in Figure 2A after filtering with *p* value < 0.05. In addition, 25 pCR samples and 76 non-pCR samples were used for the estimated immune cell content analysis (Supplementary Table S3). The correlation of 22 immune cells in the TIME is shown in Figure 2B. The correlation coefficient of naive B cells and naive CD4 T cells reached 0.47, and the correlation coefficient of naive M0 macrophages and naive CD4 T cells was −0.41. Moreover, the correlation coefficient of M0 macrophages and M1 macrophages was −0.4. Differential analysis of the 22 immune cell fractions in pCR and non-pCR samples was performed, and the results were visualized using a violin plot (Figure 2C). M1 macrophages, M2 macrophages, resting mast cells, and γδT cells had high proportions and statistically significant differences (*p* value<0.05). Among these, the levels of M1 macrophages and γδT cells were higher in the pCR samples, whereas those of M2 macrophages and resting mast cells were higher in the non-pCR samples.

Moreover, the fractions of the γδT cells, M1 macrophages, M2 macrophages, and resting mast cells obtained from the gene expression data of the GSE31519 dataset and the survival data of samples are shown in Supplementary Table S4. These four types of immune cells in the TIME were utilized to explore the EFS benefit in TNBC patients with or without NAC. An EFS benefit trend was observed in 63 TNBC patients with high M1 macrophages and γδT cells (Figure 2D), and this trend was also found in 18 patients who received NAC (Figure 2E). However, the EFS benefit trend in TNBC patients with low M2 macrophages and resting mast cells was only found in patients who received NAC.

**FIGURE 3:**
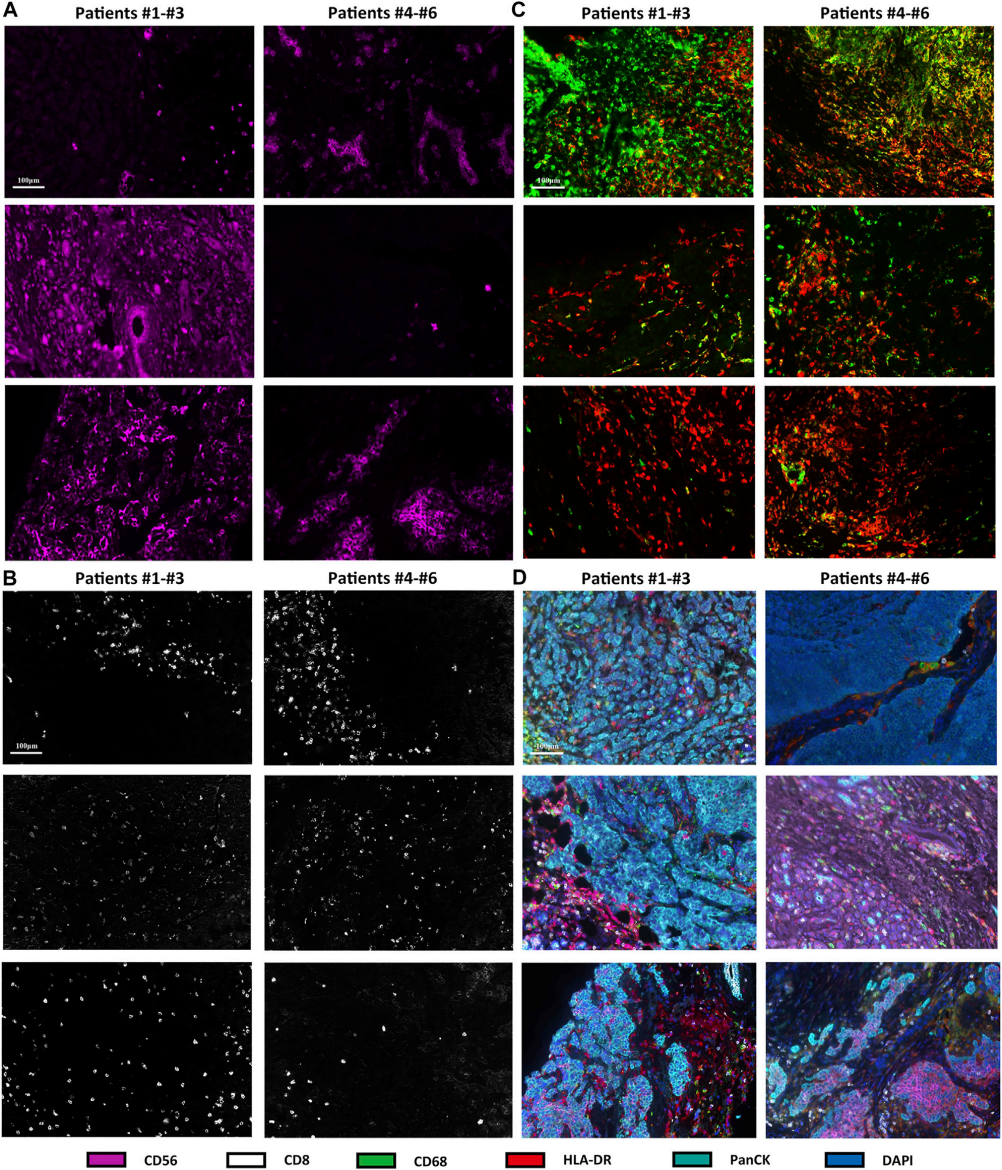
mIHC/IF results of different immune cell subtypes from six TNBC patients received NAC. The fluorescence staining of CD56 **(A)**, CD8 **(B)**, CD68, and HLA-DR **(C)**. **(D)** mIHC/IF staining results of CD56, CD8, CD68, HLA-DR, PanCK, and DAPI.

Immunofluorescence analysis of six TNBC samples after NAC showed CD56-positive natural killer (NK) cells, CD8-positive T cells, CD68-positive/HLA-DR-positive macrophages (M1 macrophages), and PanCK-positive tumor cells (Figures 3A–D). The fraction of immune and tumor cells mentioned above in each sample and the corresponding DFS are shown in Table 2. The results of the t-test to determine the difference in the fraction of cells between the short-DFS (Patients #1-3) and long-DFS (Patients #4-6) groups showed no significant differences in statistics (Supplementary Figure S2).

**TABLE 2:** Fraction of immune and tumor cells mentioned in each sample.

Sample No.	1	2	3	4	5	6
cell_count	22830	24545	26996	38035	26760	36061
Hscore_CD56 bright	1.5988	3.1697	250.5519	1.6380	2.0254	1.3394
Hscore_CD56 dim	22.1901	53.7421	72.5033	19.2849	11.1584	1.9328
Hscore_CD68	4.7919	3.3897	1.5743	67.3590	6.7975	2.4653
Hscore_CD8	2.1419	1.7315	2.3374	2.0297	4.8916	1.0815
Hscore_HLADR	50.6088	31.8476	5.9787	34.6286	18.8565	21.4747
Hscore_PANCK	71.6513	61.0226	79.1821	32.0968	77.4402	51.7484
r_CD56 bright_P	0.0012	0.0267	0.2011	0.0154	0.0199	0.0057
r_CD56 dim_P	0.0525	0.3973	0.2701	0.1490	0.0625	0.0057
r_NK	0.0537	0.4240	0.4712	0.1644	0.0824	0.0114
r_CD68_P	0.0423	0.0332	0.0142	0.2227	0.0579	0.0189
r_CD8_P	0.0138	0.0139	0.0189	0.0187	0.0330	0.0054
r_HLADR_P	0.2793	0.2224	0.0423	0.2756	0.1378	0.1332
r_CD68.HLADR_PP	0.0398	0.0262	0.0063	0.1402	0.0341	0.0177
r_CD68.HLADR_PN	0.0025	0.0070	0.0079	0.0825	0.0238	0.0012
M1/macrophage	0.9420	0.7892	0.4424	0.6296	0.5888	0.9384
r_PANCK_P	0.3760	0.3534	0.4874	0.2464	0.4037	0.3726
IM/tumor	0.2919	1.3332	1.0347	1.6469	0.4293	0.0957
DFS (month)	5	5	7	23	13	14

M1/Macrophage, r_CD68.HLADR_PP/r_CD68_P; IM/Tumor, (r_NK + r_CD68_P + r_CD8_P)/r_PANCK_P; DFS, disease-free survival.

### Changes in Immune-Related Genes and Immune Cell Fractions in BC Patients Before and After NAC

**FIGURE 4:**
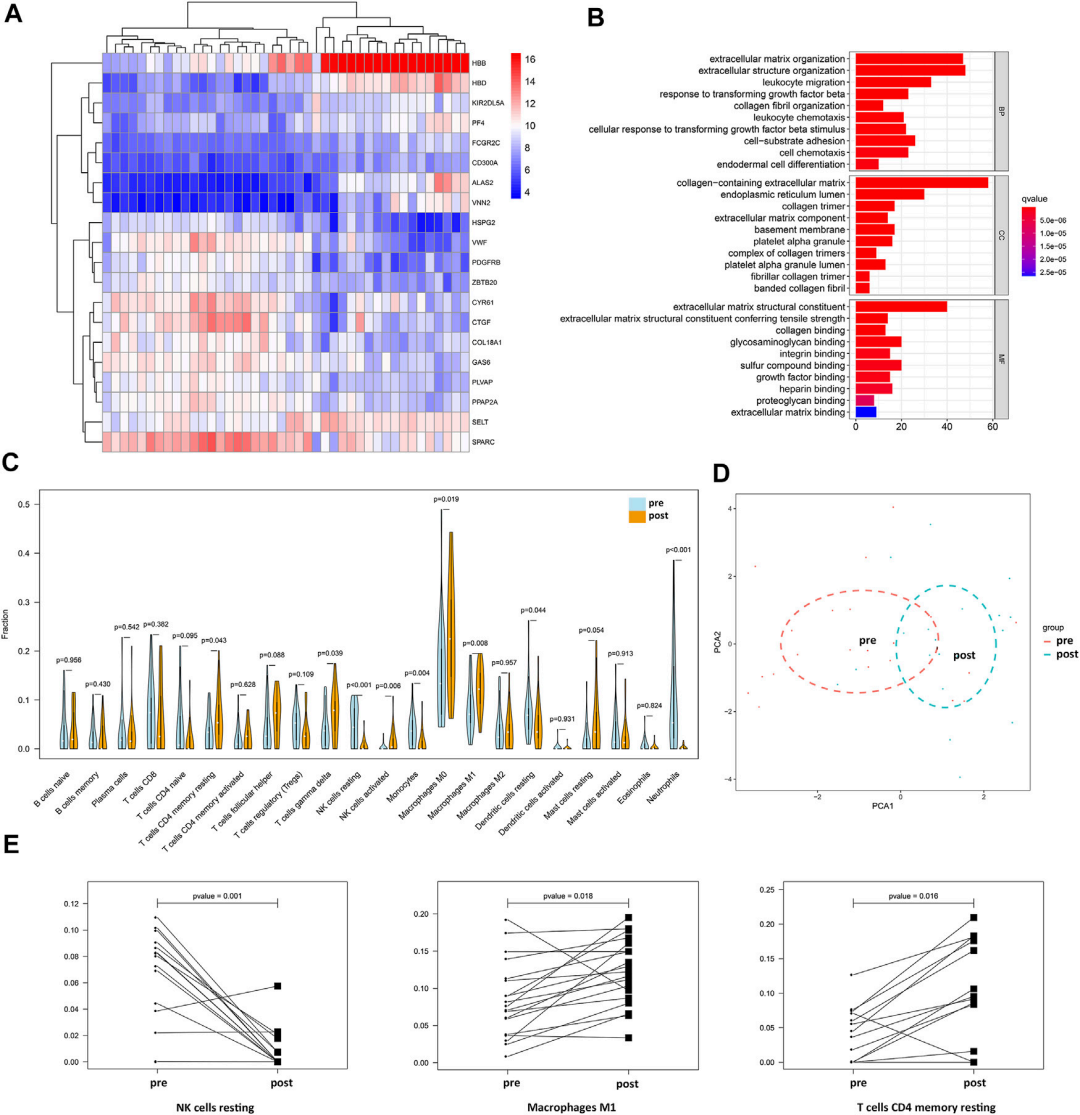
Changes in immune-related genes and immune cell fractions in BC patients before and after NAC. **(A)** Genetic clustering analysis of the heatmap showed DEGs (*p* value < 0.05, logFC >1 or logFC < −1) with the fold change TOP20 from dataset GSE32072. **(B)** BP, CC, and MF of DEGs from dataset GSE32072. **(C)** Differential analysis of 22 immune cell fractions in pre-NAC and post-NAC samples from dataset GSE32072. Blue represents pre-NAC samples and orange represents pre-NAC samples, and *p* value represents the statistical significance of the differential analysis between the two groups for each immune cell. **(D)** PCA cluster analysis showed that the red dots represent the sample before NAC and the blue dots represent the sample after NAC. **(E)** Paired difference analysis of resting NK cells and M1 macrophages from dataset GSE32072 and CD4 memory resting T cells from dataset GSE18728.

In total, 21 samples before NAC and the corresponding paired samples after NAC were used for comparisons of gene expression in the GSE32072 dataset, which identified 352 DEGs (filtered with adjusted *p* value = 0.05 and logFC = 1; Supplementary Table S5), including immune-related genes, such as *FCGR2C*, *KIR2DL5A*, and *CD300A*. Genetic clustering analysis of the heatmap showed the top 20 genes with an absolute value of logFC (Figure 4A). In addition, GO enrichment analysis showed that the DEGs were mainly enriched in BPs that included migration and chemotaxis of leukocytes and extracellular matrix organization, and the MF of the DEGs was to regulate the activity of extracellular matrix binding (Figure 4B).

From the gene expression data of samples before and after NAC in the GSE32072 dataset, the 22 immune cell type fractions were obtained after filtering with a *p* value < 0.05. Moreover, 21 pre-NAC samples and 19 post-NAC samples were utilized to estimate immune cell content (Supplementary Table S6). Differential analysis of 22 immune cell fractions before and after NAC samples was performed to obtain a violin plot (Figure 4C). M1 macrophages, γδT cells, resting CD4 memory T cells, resting natural killer (NK) cells, and resting dendritic cells were found to be among the 22 types of immune cells with high proportions and statistically significant differences (*p* <0.05). Among these, the levels of M1 macrophages, γδT cells, and resting CD4 memory T cells were higher in post-NAC samples, whereas those of resting NK cells and resting dendritic cells were higher in pre-NAC samples. PCA with cluster analysis was performed using the differences in the 22 immune cell fractions in the GSE32072 dataset (Figure 4D). In the PCA, the samples before and after NAC were not completely separated, but the trend of dispersion was observed. After filtering with a *p* value < 0.05, 19 pre-NAC samples and corresponding post-NAC samples were utilized to estimate the immune cell content from data in the GSE18728 dataset (Supplementary Table S7). The paired difference analysis of resting NK cells (from the GSE32072 dataset), M1 macrophages (from the GSE32072 dataset), and resting CD4 memory T cells (from the GSE18728 dataset) before and after NAC is shown in Figure 4E. These findings demonstrated that the levels of M1 macrophages and resting CD4 memory T cells were higher in post-NAC samples, whereas those of resting NK cells were higher in pre-NAC samples.

## Discussion

In the present study, we identified several immune-related genes that correlated with the efficacy of NAC in breast cancer patients. In contrast to other prognostic-related signatures, such as survival prognostic subnetwork signatures (SPNs) (Song et al., 2015), the present study focused on breast cancer patients who received NAC, and the differential expression of immune-related genes was used to identify a correlation between NAC and immune effects. Four of the immune-related genes were chemokines, namely, *CXCL1*, *CXCL9*, *CXCL10*, and *CXCL11*, and the other genes were *IDO1*, *IFNG*, and *ORM1*. Chemokines secreted by cancer cells are a class of molecules involved in shaping the TIME and regulating the balance between antitumor and protumor factors, which induces leukocytes to enter the TIME and indirectly induces the tumor-related stromal compartment to secrete angiogenic and lymphangiogenic growth factors (Unver, 2019). Among them, *CXCL9*, *CXCL10*, and *CXCL11* promote the antitumor immune response by recruiting Th1, CD8^+^ T, and NK cells in the TIME (Nagarsheth et al., 2017), which is consistent with the enriched pathways of leukocyte chemotaxis and migration in the present study. Notably, *CXCL9* and TILs have been shown to be highly significant predictors of NAC responses in classic studies, such as the GeparDuo (Denkert et al., 2010) and GeparSixto (Denkert et al., 2015) trials. In addition, the immunoregulatory effect of *CXCL9*, *CXCL10*, and the *CXCL11/CXCR3* axis on tumor sites to promote antitumor immunity has been used as a new tumor therapeutic target (Tokunaga et al., 2018). Not only positive results of *CXCR3* antagonists *in vitro* and *in vivo* in the preclinical model of BC have been obtained, but also positive results of various immune checkpoint inhibitors (ICIs) regulating *CXCL9*, *CXCL10*, and *CXCL11* in clinical studies have been obtained (Karin, 2018).

*CXCL1* generally promotes tumor progression in melanoma, colorectal cancer, breast cancer and other tumors. A previous study (Franklin et al., 2020) has reported that in patients with TNBC, Ras/MAPK-related gene expression signatures positively correlate with myeloid/neutrophil-recruiting *CXCL1/2* expression and negatively correlate with *CXCL9/10/11*. However, another study (Lv et al., 2014) has reported that *miR141-CXCL1-CXCR2* signaling–induced Treg recruitment regulates the metastasis and survival of non–small cell lung cancer; therefore, the type of immune cells that *CXCL1* recruits in the TIME should be further explored. Interestingly, the expression of *IL-17A* and *CXCL1* mRNA is increased in BC cells, and their changes are correlated (Ma et al., 2018), indicating that Th17 cells are increased in CXCL1-induced BC progression. Thus, *CXCL1* combined with the IL-17 signaling pathway should be further investigated.

Indoleamine 2,3-dioxygenase 1 (*IDO1*) has been used as an immunotherapy target and prognostic immune-related biomarker in recent years. Contrary to the positive correlation between *IDO1* and TILs in most studies (Zhai et al., 2015) and the expression of *IDO1* indicating a good NAC response in BC, other studies (Salvador-Coloma et al., 2020) have shown that *IDO1* is positively correlated with early myeloid–derived suppressor cells (eMDSCs) and has a poor NAC response, which may be related to the different influences of *IDO1* expression in tumor cells and immune stromal cells on prognosis. *IFNG* is an IFN-γ–encoding gene that plays an orchestrating role in innate immunity and inflammatory responses in the TIME. In the TIME of BC, M1 macrophages, CD8^+^ T cells, dendritic cells (DCs), and neutrophils all inhibit tumor growth by secreting inflammatory cytokines, such as IFN-γ (Shihab et al., 2020). As an acute phase protein, *ORM1* has the ability to induce monocytes to release IL-1, TNF-α, IL-6, and IL-12 (Ligresti et al., 2012), and a multivariate classification model using the six proteins, including ORM1, predicts responses to NAC and further predicts relapse-free survival of BC patients (Hyung et al., 2011).

In the present study, the expression of the abovementioned genes was higher in the group with a good response to NAC, which could select patients who would respond more efficiently to NAC and further explain the significance of immunotherapy in addition to chemotherapy in NST for BC patients with these gene signatures. Furthermore, the expression of immune-related genes, such as *FCGR2C*, *KIR2DL5A*, and *CD300A*, was found to change before and after NAC in the present study. These genes are all downregulated after NAC and are related to the killing efficacy of NK cells (van der Heijden et al., 2012; De Re et al., 2014; Lopez-Sejas et al., 2016). These findings not only suggest that local immune downregulation occurs in the TIME after NAC but also reiterate the necessity of adding immunotherapy to NST to modify the TIME to achieve better efficacy in BC.

For the interaction between immune cells in the TIME and NAC in BC, the close correlation between the 22 immune cell fractions may reflect crosstalk in the TIME, which was the basis for the hypothesis for observing the difference in the TIME between tumor samples with or without response to NAC in the present study. Interestingly, the different immune cell subtypes in TILs were associated with different responses to NAC in BC patients in the present study. Among these immune cells, the levels of M1 macrophages and γδT cells were higher in the pCR samples, whereas those of M2 macrophages and resting mast cells were higher in the non-pCR samples, which was consistent with the results of previous studies (Kaewkangsadan et al., 2017; Reddy et al., 2019), showing the correlation between NAC response and immune cells. However, the γδT cells have been shown to be associated with immunosuppression in the TIME of BC and poor prognosis (Morrow et al., 2019). Such inconsistency may be related to the different functions of the γδT cell subtypes, but the unique antigen-recognition ability of γδT cells in immunotherapy has shown promise in the combination of NAC and immunotherapy (Fisher et al., 2014). A previous study (Patin et al., 2018) has shown that type I IFN receptor signaling controls the activity of protumoral *IL17A*-producing γδT cells in breast cancer, which is consistent with the high expression of IFNG, the high proportion of the γδT cells in the pCR group, and the enriched IL-17 signaling pathway in the present study. According to the results in the TCGA database, high expression levels of *CXCL1*, *CXCL9*, *IDO1*, and *IFNG* were correlated with higher proportions of M1 macrophages, CD8 T cells, and resting CD4 memory T cells, but low expression levels of these genes were correlated with lower proportions of M2 macrophages and mast cells. The expression of these immune-related genes indicated the immune activation state of the TIME, and the abovementioned results were consistent with the role of these immune cells in the TIME (Ravelli et al., 2017). Except for the correlation between the proportion of different immune cells and pCR rate, the prognosis-related TIME of breast cancer, especially that of the TNBC patients who received NAC, was emphasized by the results of the EFS benefit with different immune cell components in the GSE31519 dataset.

In the present study, the changes in the TIME induced by NAC were reflected by increased M1 macrophages, γδT cells, and resting CD4 memory T cells but decreased NK cells and DCs. However, previous studies analyzing TILs before and after changes in clinical BC samples have reported contradicting results (Kim et al., 2020; Wesolowski et al., 2020). Thus, it remains unknown which immune cells will increase or decrease after NAC. However, as long as the immune cell subtype in the TIME changes after NAC, it will provide the basis for NAC combined with immunotherapy and the corresponding target cells. Modulating the TIME can be achieved either by targeting immune checkpoints on the increased immune cells or by avoiding apoptosis of the decreased immune cells, which is a method for immunotherapy to achieve better therapeutic effects in combination with NAC. More importantly, previous studies (Hamy et al., 2019; Herrero-Vicent et al., 2019) have shown that the increase in TILs after NAC is correlated with shorter DFS and aggressive tumor characteristics, suggesting the importance of TILs before and after NAC as a prognostic biomarker. Consistent with this finding, our mIHC/IF results showed that patients #4–6 with fewer total NK cells and more M1 macrophages had longer DFS after NAC. These results suggested that different subtypes of TILs had the potential of indicating different DFS outcomes in TNBC after NAC, thus highlighting the significance of combining NAC with ICIs targeting different immune cells to achieve better clinical benefits. However, due to the small sample size in the present study, our results were not statistically calculated. One limitation of the present study was that the changes in immune cells in samples before and after NAC were not compared due to sampling limitations. In the future, our team will expand the sample size and continue to explore the temporal and spatial heterogeneity of the TIME before and after NAC in BC. In addition, the relationship between the changes in peripheral blood immune cells and TILs is currently a hot topic of research as there is an urgent need for dynamic monitoring of biomarkers to screen appropriate immunotherapy populations for BC patients (Axelrod et al., 2020).

## Conclusion

Immune-related genes and different subtypes of immune cells are biomarkers correlated with the therapeutic effect of NAC for BC. In the present study, the interaction between the TIME and NAC was discussed in terms of the therapeutic effect of different TIMEs on chemotherapy and the changes in the TIME after NAC. Several immune-related genes and immune cell subtypes related to the NAC response were identified, and several immune cell fractions were significantly changed before and after NAC. The present study analyzed samples in databases and clinics to conduct a more detailed study on the correlation between the efficacy of NAC in BC and the TIME, which further verified the importance of immunotherapy combined with chemotherapy.

## Data Availability

The datasets presented in this study can be found in online repositories. The names of the repository/repositories and accession number(s) can be found in the article/Supplementary Material.

## Supplementary Material

Supplementary Figure S1 Differences between immune cell fractions of the TIME in BC patients with different expressions of DEGs. The data from the TCGA database showed that the expressions of immune-related DEGs we extracted from GSE32646 are associated with 22 immune cell fractions: Green is for low-expression samples and red is for high-expression samples, and *p* value represents the statistical significance of the differential analysis between the two groups for each immune cell. **(A)** Expression of CXCL1. **(B)** Expression of CXCL9. **(C)** Expression of IDO1. **(D)** Expression of IFNG. **(E)** Expression of ORM1.

Supplementary Figure S2 Differences in the fraction of cells between the short-DFS and long-DFS groups. T-test differential analyses in the fraction of cells between the short-DFS (Patients #1–3) and long-DFS (Patients #4–6) groups.

Supplementary Table S1 Detailed information about the five datasets.

Supplementary Table S2 302 DEGs between the pCR group and non-pCR group from the dataset GSE32646.

Supplementary Table S3 Immune cell content analysis from the dataset GSE32646.

Supplementary Table S4 Immune cell content analysis and the survival data from GSE31519.

Supplementary Table S5 352 DEGs between before and after NAC groups from GSE32072.

Supplementary Table S6 Immune cell content analysis from the dataset GSE32072.

Supplementary Table S7 Immune cell content analysis from the dataset GSE18728.
