# Supplementary material for: Correlation Between Immune-Related Genes and Tumor-Infiltrating Immune Cells With the Efficacy of Neoadjuvant Chemotherapy for Breast Cancer
Source: Front Genet. 2022 Jun 8;13:905617. doi: 10.3389/fgene.2022.905617 (PMC9214242; doi:10.3389/fgene.2022.905617)
Supplement: Supplementary file 3 [file Table1.DOCX]

| Datasets | GSE123845 | GSE32646 | GSE31519 | GSE32072 | GSE18728 |
| --- | --- | --- | --- | --- | --- |
| Experiment type | Expression profiling by high throughput sequencing | Expression profiling by array | Expression profiling by array | Expression profiling by array | Expression profiling by array |
| Sample size | 136 | 115 | 67 | 42 | 39 |
| Experimental groups | 89 non-pCR samples after NAC | 88 non-pCR samples after NAC | the samples with low proportion of different kinds of immune cells | 21 samples after NAC | 19 samples after NAC |
| Control groups | 47 samples achieved pCR after NAC | 27 samples achieved pCR after NAC | the samples with high proportion of different kinds of immune cells | 21 samples before NAC | 20 samples before NAC |
| Drugs used in NAC | AC+T\AC+TH\TCHP | P-FEC | 18 of 67 received NAC, drugs not detailed | AT | Docetaxel and Capecitabine |
| pCR, pathological complete response; NAC, neoadjuvant chemotherapy; AC, doxorubin/cyclophosphamide; T, paclitaxel; TH, paclitaxel/trastuzumab; TCHP, paclitaxel/carboplatin/trastuzumab/pertuzumab; P-FEC, paclitaxel followed by 5-fluorouracil/epirubicin/cyclophosphamide;AT, doxorubicin plus paclitaxel. | | | | | |

Supplement Table1 Information about the datasets and descriptions of the control and experimental groups for each dataset
