# Supplementary figures and images for: Correlation Between Immune-Related Genes and Tumor-Infiltrating Immune Cells With the Efficacy of Neoadjuvant Chemotherapy for Breast Cancer
Source: Front Genet. 2022 Jun 8;13:905617. doi: 10.3389/fgene.2022.905617 (PMC9214242; doi:10.3389/fgene.2022.905617)

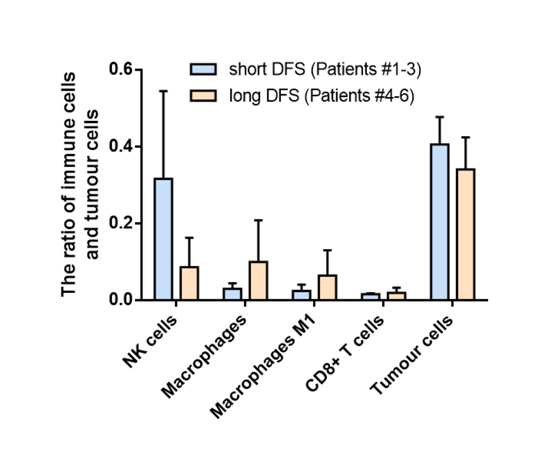

Supplement: Supplementary file 4 [file Image2.TIF]

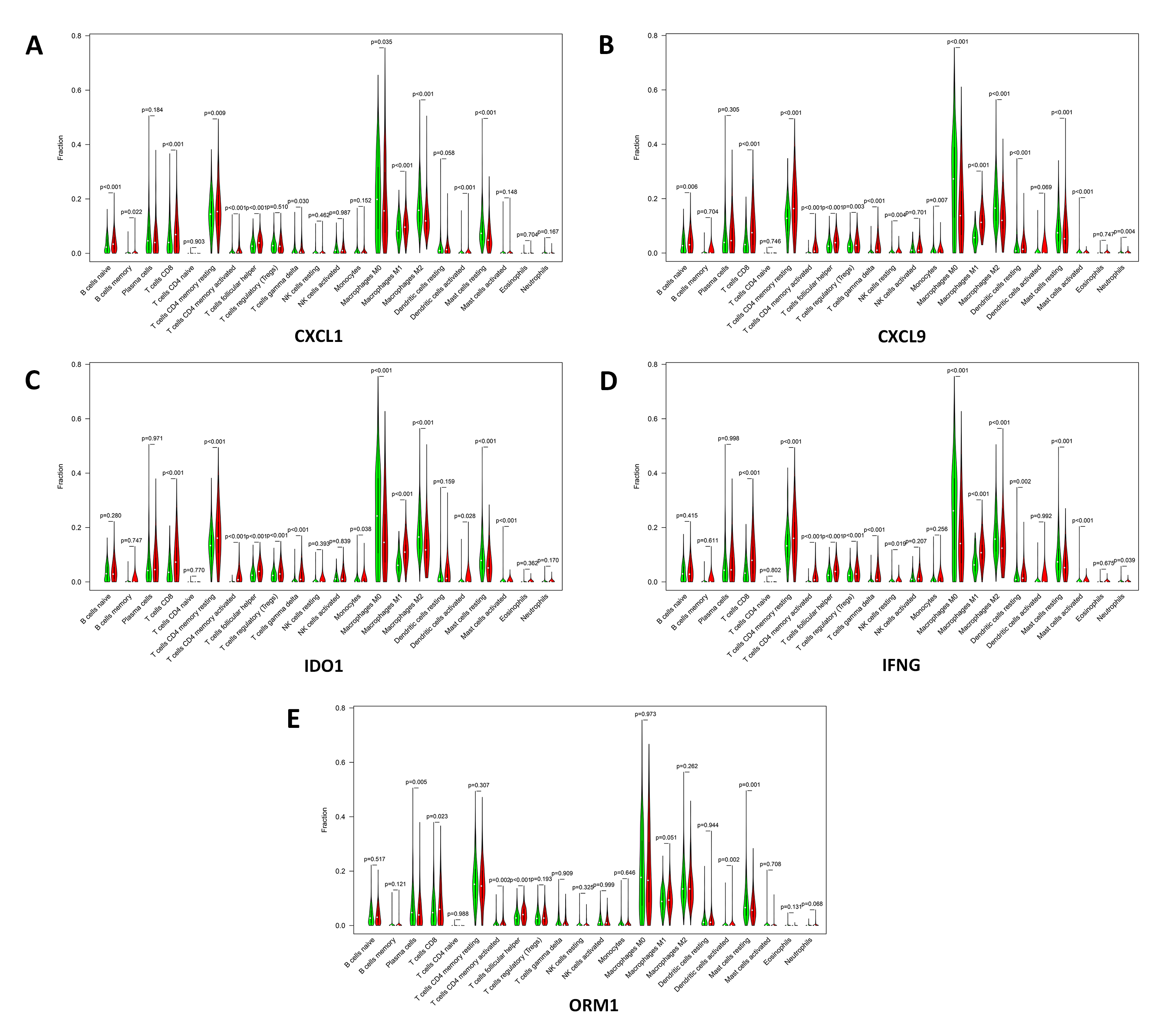

Supplement: Supplementary file 5 [file Image1.TIF]
